# Error in Citation

**DOI:** 10.1001/jamanetworkopen.2026.13760

**Published:** 2026-04-24

In the Invited Commentary titled “Delayed Diagnosis of Ovarian Cancer and the Association With Disease Outcomes,”^1^ published March 27, 2026, the first author was misidentified. This should have appeared as Soppe et al.^1^ This article has been corrected.^1^
